# Dental Biofilm and Laboratory Microbial Culture Models for Cariology Research

**DOI:** 10.3390/dj5020021

**Published:** 2017-06-19

**Authors:** Ollie Yiru Yu, Irene Shuping Zhao, May Lei Mei, Edward Chin-Man Lo, Chun-Hung Chu

**Affiliations:** Faculty of Dentistry, The University of Hong Kong, Hong Kong, China; yuyiru@hku.hk (O.Y.Y.); irenezhao110@gmail.com (I.S.Z.); mei1123@hku.hk (M.L.M.); hrdplcm@hkucc.hku.hk (E.C.-M.L.)

**Keywords:** biofilm, dental plaque, demineralization, remineralization, caries, review

## Abstract

Dental caries form through a complex interaction over time among dental plaque, fermentable carbohydrate, and host factors (including teeth and saliva). As a key factor, dental plaque or biofilm substantially influence the characteristic of the carious lesions. Laboratory microbial culture models are often used because they provide a controllable and constant environment for cariology research. Moreover, they do not have ethical problems associated with clinical studies. The design of the microbial culture model varies from simple to sophisticated according to the purpose of the investigation. Each model is a compromise between the reality of the oral cavity and the simplification of the model. Researchers, however, can still obtain meaningful and useful results from the models they select. Laboratory microbial culture models can be categorized into a closed system and an open system. Models in the closed system have a finite supply of nutrients, and are also simple and cost-effective. Models in the open system enabled the supply of a fresh culture medium and the removal of metabolites and spent culture liquid simultaneously. They provide better regulation of the biofilm growth rate than the models in the closed system. This review paper gives an overview of the dental plaque biofilm and laboratory microbial culture models used for cariology research.

## 1. Introduction

Dental caries is the localized destruction of dental hard tissues by acidic byproducts from dental plaque containing acid-producing bacteria. Cariology research allows the investigation of caries’ pathogenicity, testing the effects of new caries-prevention methods (i.e., some devices and drugs) and developing new caries-preventing products. This review paper gives an overview of the dental plaque biofilm and in vitro biofilm models used for cariology research. It aims to provide essential and instructive information for researchers who seek to plan and design cariology research.

## 2. The Dental Plaque Biofilm

**Figure 1:**
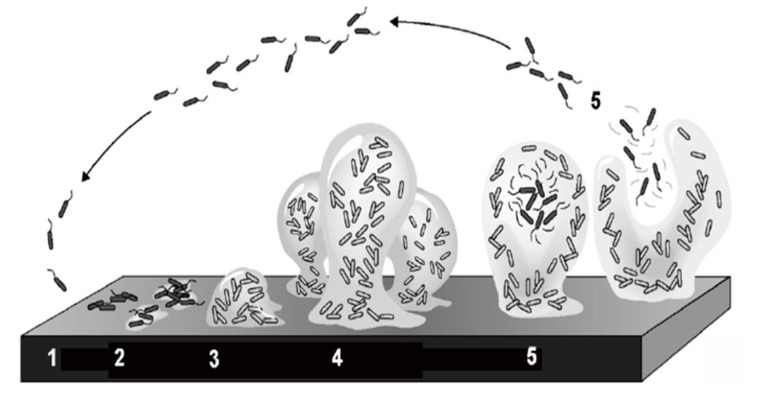
Five stages of biofilm formation and growth (adapted from Stoodley et al., 2002 [2], with permission from © 2002 Annual Reviews Directory. License number: 4131221128126).

Dental plaque is an oral microbial biofilm that is found on exposed tooth surfaces in the mouth. It has a large diversity of species and consists of densely packed bacteria embedded in a matrix of organic polymers of bacterial and salivary origin. Dental plaque is the causal agent of dental caries in the presence of sugar and time. In the oral cavity, the formation of dental plaque on the tooth surface follows a similar sequence to that of biofilms in other natural ecosystems. A biofilm is formed by bacteria sticking to each other and, often, adhering to a surface. The bacteria are embedded within a self-produced matrix of extracellular polymeric substance. In dental biofilm, *streptococcus mutans* is a major bacterium producing the extracellular polysaccharide matrix in dental biofilms. The bacterial cells growing in a biofilm are physiologically distinct from planktonic cells which float or swim in a liquid medium. Bacteria in the plaque biofilm can respond to many factors, such as cellular recognition of specific or non-specific attachment sites on a surface and nutritional signals. Marsh and Martin [1] divided the formation and growth of oral biofilm into five stages (Figure 1).

Oral biofilms can form on almost any surface present in the oral cavity including enamel, dentin, cementum, gingiva, oral mucosa, carious lesion, restoration, dental implant, and denture. Dental plaque will colonize rapidly, not only the coronal enamel surface but also the exposed root surface. The growth of microbiota on the exposed root surface proceeds more rapidly than that on the smooth enamel surface because of the irregular surface topography of the exposed root dentin surface. The organization and structure of dental plaque vary considerably according to the sites where plaque forms [1]. The growth of microorganisms on specific oral niches is affected by various factors such as acidity (pH) of the environment, availability of nutrients, presence of antimicrobial agents, and host defense.

Surface-bound microorganisms have a survival and/or selective advantage over their planktonic phases [1]. Bacteria in dental plaque have stronger resistance to antimicrobial agents than planktonic bacteria. Bacterial extracellular polysaccharides prevent the perfusion of antimicrobial agents to bacterial targets; this acts as a barrier to protect the plaque bacteria against certain environmental threats such as antibiotics, antibodies, surfactant, bacteriophage, and white blood cells [3]. Resistance of biofilm bacteria to antimicrobial agents may also develop. As a result, the minimum inhibitory concentration of antimicrobial agents against bacteria in biofilm is significantly higher (up to 1000-fold) than that in liquid [1].

Though there are many bacteria associated with dental caries, a few groups of cariogenic bacteria such as *streptococci*, *actinomycetes,* and *lactobacilli* are found to be more closely associated than the others. These groups of bacteria often dominantly proliferate in the dental biofilm collected from the carious lesions of teeth. *Streptococcus* is the predominant species in cariogenic microbe. It colonizes clean tooth surfaces at an early stage, and it also relates to root caries. The predominant coccal isolated from carious dentin in root caries are *S. mutans*, *S. sanguis*, and *S. mitis* [4]. *S. mutans* and *S. sobrinus* are difficult to distinguish. Hence, these two species are always lumped together and regarded as *mutans streptococci*. *Mutans streptococci* can adapt to acidic environments, which is the key factor contributing to its cariogenic potential. *Actinomycetes* is an initial colonizer of human root surfaces. *A. naeslundii* and *A. viscosus* can induce root surface caries [5]. *Actinomycetes* is often isolated from subgingival microflora and from plaque associated with root caries [6] (they have long surface appendages named fibrils, or fimbriae). The fibrils allow *actinomycetes* to adhere to the surface of tooth roots. Fibrils also improve the attachment of *actinomycetes* to other bacteria in dental plaque. *Lactobacilli* are aciduric bacteria, including *L. acidophilus*, *L. rhamnosus*, *L. casei*, and *L. oris* [7]. Patients with caries have higher counts of *lactobacilli* than those with no caries. Evaluating the amount of *Lactobacilli* in saliva is used as a caries-activity testing method in clinical assessment [8]. *Lactobacilli* is difficult to grow and mature as a mono-species biofilm. However, it can be a predominate species in a substantial biofilm in the presence of *S. mutans* [9]. A potential relationship was found among some species of *lactobacilli*, *streptococci,* and *actinomycetes* in the root caries formation process [10].

## 3. Laboratory Microbial Culture Models

Laboratory microbial culture models simulate the oral environment for cariology study. Unlike in vivo studies, they do not have problems relating to the uncontrollable fluctuating locus-specific of the oral environment [11,12]. Two complementary microbiological approaches can be taken to generate biofilm in microbial culture models. The first is the evolution of a plaque microcosm from natural oral microflora. A microcosm is defined as “a laboratory subset of the natural system from which it originates and from which it also evolves” [13]. Microcosm plaques are similar in composition, growth, acidity (pH) behavior, biochemical properties, and (probably) in complexity to natural plaque.

The second approach is the construction of defined-species biofilm consortia with major plaque species, or a mixture of different species of the acquired oral bacteria (such as the American Type Culture Collection (ACTT) bacteria). Consortia are simpler than plaque microcosms; they have the advantage of incorporating individual bacterial species. Even in a simple batch culture method, oral multispecies consortia can develop complex biofilms on enamel and dentin that can induce carious lesions similar to those in vivo. The designs of laboratory microbial culture models vary according to the purpose of the laboratory studies. They can be classified as closed system and open system. Each system is a compromise between the reality of the in vivo ecosystem and the simplification of the system. However, a well-designed model and study allow researchers to obtain meaningful and useful results [13].

### 3.1 The Closed System

Microbial culture models in the closed system have a finite supply of nutrients. The growth rates of the biofilm are rapid at the beginning of the cultivation when there are ample nutrients. However, this is uncommon in the natural growth of biofilm [14,15]. The growth conditions will change considerably with consumption of the nutrients and the accumulation of metabolic products. Hence, the physiological and biological properties of the biofilm are not comparable with the natural ones. Researchers used closed system models because of their simplicity, high productivity, repeatability, controllability of the experimental conditions, less contamination, and cost-effective properties. The agar plate and microtiter biofilm models are two examples of the common microbial culture models in closed system.

#### 3.1.1. The Agar Plate

**Figure 2:**
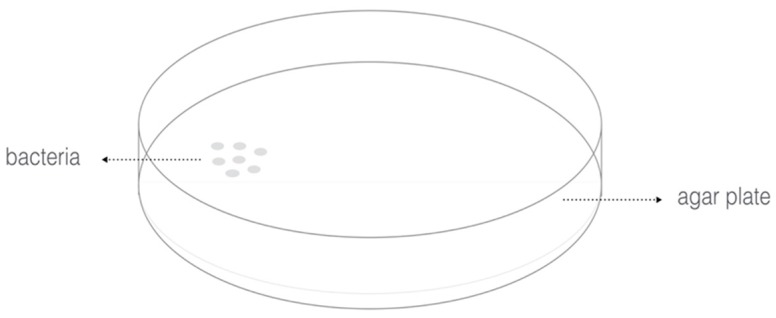
Agar plate.

The agar plate is one of the simplest laboratory microbial culture models (Figure 2). The nutrient supply is not continuous. Bacteria growth on the surface of the agar can only be supported until the finite nutrient is exhausted. Thus, results of studies using this simplistic model should be interpreted with caution. This situation is different from bacterial growth on a hard tissue surface, because the biofilm consumes nutrients from the substrate. It resembles biofilms associated with soft tissue infections or growing in an extracellular matrix. This model has been used to test the susceptibility of oral biofilm to various antimicrobials, especially some light active chemicals [16,17]. The disc-diffusion method is not an ideal way to predict the therapeutic effects of antimicrobial [18]. The effects of the antibacterial agents can be misinterpreted because the cationic antibacterial agents may combine with the anionic agar polysaccharide gel [19].

#### 3.1.2. The Microtiter Biofilm Model

**Figure 3:**
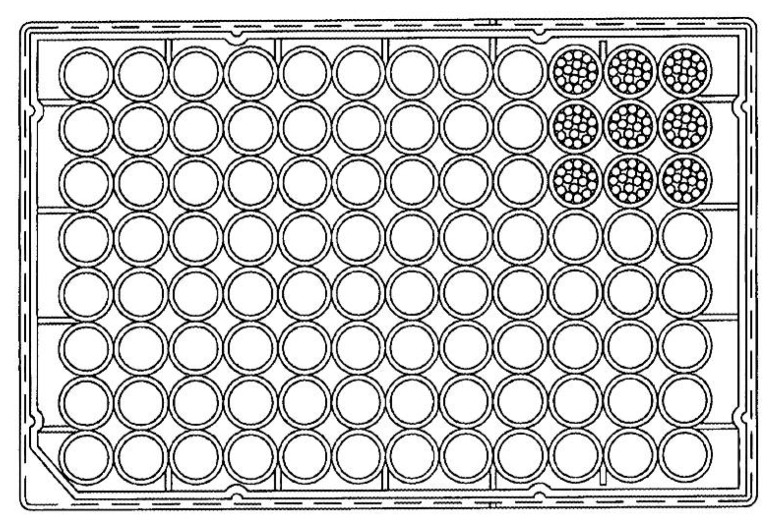
Configuration of the 96-well microtiter.

The microtiter biofilm model is made of a multiple-well microtiter plate. A microtiter plate is commonly made of polystyrene, but it can be manufactured in a variety of materials. A microtiter plate is a flat plate with multiple “wells” (used as small test tubes). A standard definition of a microtiter plate was developed by the Society for Laboratory Automation and Screening (SLAS) and published by the American National Standards Institute (ANSI). Henceforth, the microplate standards are known as ANSI/SLAS standards. A configuration of a 96-well microtiter is shown in Figure 3. Each well of a microplate typically holds several milliliters of liquid. The microplate is regarded as a standard tool in cariology research, allowing the biofilm to grow independently in each well.

### 3.2. The Open System

The open system can be described as a continuous culture system. It enables the supply of a fresh culture medium and the removal of metabolites and spent culture liquid simultaneously. Hence, the concentration of bacteria and metabolic products remains constant [20]. Moreover, the biofilms can stay in a stable state or keep in a dynamic balance [21]. Nevertheless, the repeatability of the experimental result is low because of the heterogeneity of the biofilm in the open system. Besides, the possibility of contamination can be high due to the complexity of the construction.

The open system simulates the in vivo environment better than the closed system. It also allows better regulation of the biofilm growth rate and other variables. Common microbial culture models in the open system include the chemostat model, the flow cell biofilm model, the constant depth film fermenter model, the drip flow biofilm reactor, the multiple Sorbarod model, and the multiple artificial mouth model.

#### 3.2.1. Chemostat

**Figure 4:**
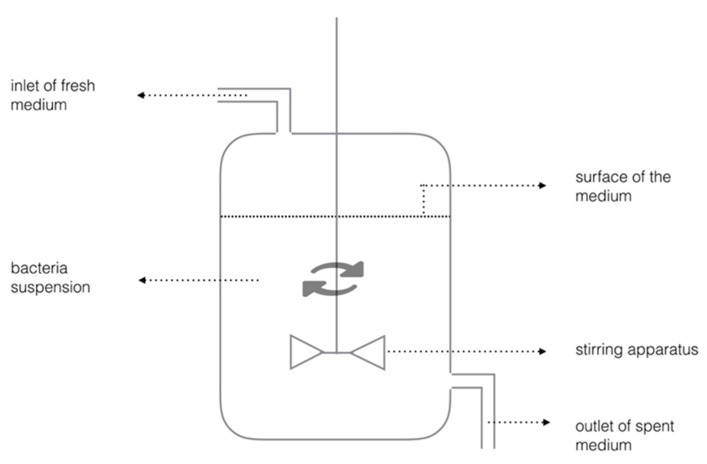
Schematic diagram of chemostat.

Chemostat is preferred for biofilm experiments because the continuous culture of chemostat can provide homogeneity and a steady environment (Figure 4). The experimental parameters can be investigated independently in the highly-controlled conditions [22]. Oral bacteria grow planktonically in a conventional chemostat. A fresh cultural medium is provided at the same rate as the culture waste liquid removal rate. Planktonic bacteria have the tendency to form biofilm at a solid-liquid interface in a chemostat. A substrate such as a tooth slice can be suspended in the chemostat to provide a surface for bacterial colonization and biofilm or dental plaque formation. Chemostat is generally expensive and space-consuming in laboratory. Precaution is needed to prevent excessive bacteria growth in chemostat, which can block the tubing [23].

#### 3.2.2. The Flow Cell Biofilm Model

**Figure 5:**
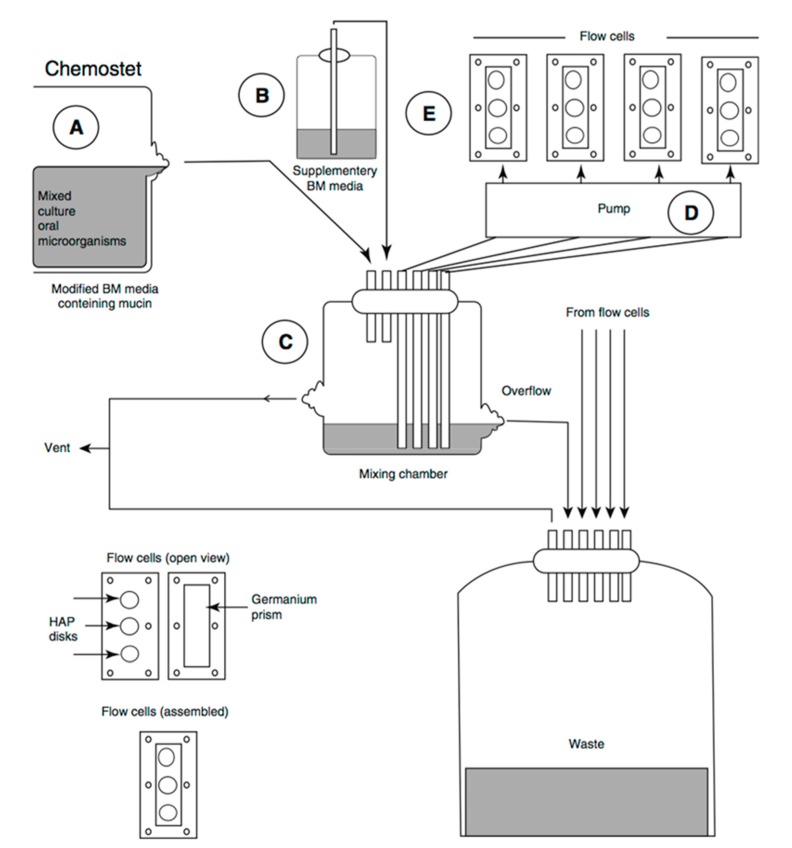
Configuration of the flow cell biofilm model (adapted from Herles et al., 1994 [28], with permission from © 1994 International & American Associations for Dental Research. License number: 4131180504654).

The flow cell biofilm model is used as perfusion chambers to observe the initial growth and physiology of stationary bacterial cells [24]. The culture fluid passes through a tube and biofilms are cultured in a flow reactor where the substratum is placed. Biofilms can grow on the surface of tooth blocks [25], microscopy glass slides, or glass rods [26]. The flow cell biofilm model is shown in Figure 5. Bacteria suspension stored in a chemostat (A) and bacteria-free medium (B) are stirred or pumped (D) to a mixed chamber (C) and go through the flow reactor (E) to create a flow. Therefore, the shear force will work on the microbe when the culture fluid passes through the surface of the biofilm. The outside chemostat in the flow cell biofilm model allows external biofilm growth, which means the growth condition can be controlled and the biofilm can grow for an extended period. Other advantages are flexibility of sample configuration, presence of fluid dynamics, plaque monitoring. and the possibility of extra experimental treatments.

The flow cell biofilm model simulates the in situ situation of undisturbed biofilm communities. The constant environment is provided with laminar flow [24]. The model has been adopted frequently in the evaluation of the effects of antimicrobial agents because it is convenient to make comparisons of viability of microbes among different experimental groups [27]. In addition, the continuous flow system simulates the clearance of antimicrobial agents in the mouth. A limitation of this device is that the laminar fluid flows through the biofilm instead of across its surface. It mimics the flow of saliva on the surface of mucosal, but the pathways of saliva flowing on hard-surface biofilms are different. Flow cell biofilm models are also expensive and space consuming.

#### 3.2.3. The Constant Depth Film Fermenter Model

**Figure 6:**
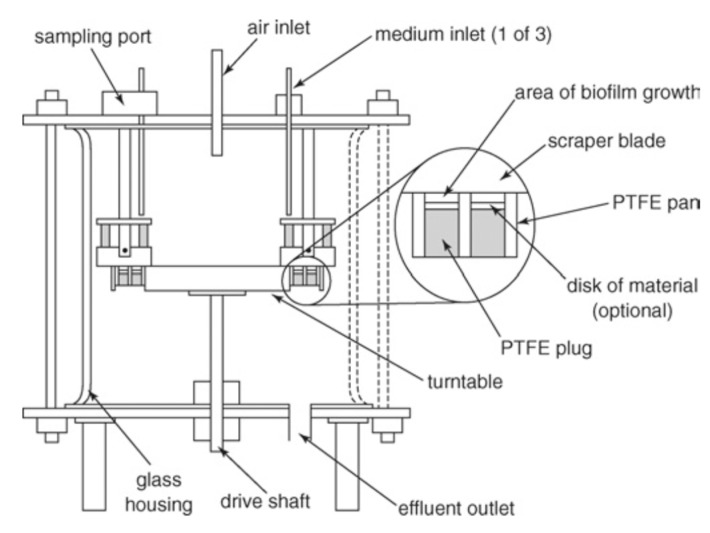
Configuration of the constant depth film fermenter (adapted from Pratten et al., 2007 [35], with permission from © 2007 Wiley Online Library. License number: 4131200175687).

The major components of the constant depth film fermenter (CDFF) model are plugs, a rotating stainless steel disk, and static scarper blades [23]. The plugs allow the growth of biofilm. The rotating stainless steel disk holds the samples. The static scarper blades control the depth of the biofilm. These components are put into a glass container where a fresh cultural medium is provided and culture waste liquid is removed. The configuration of CDFF is shown in Figure 6.

The thickness of biofilms is controlled to a predetermined depth by mechanically removing the excess biofilm. This simulates the tongue movement over the teeth. The thickness of biofilms can be 200 µm [29,30] to mimic dental plaques. The properties of biofilms that are developed are relatively constant over time. The CDFF model supports restrained growth and produces a number of replicate biofilms. Since the thickness of the biofilms is predetermined, subsampling and effluent analysis are limited to some extent [31]. The model was used to study etiology of caries [32], to assess antimicrobial effect on biofilm [33], and to investigate the structure of biofilm [34].

#### 3.2.4. The Drip Flow Biofilm Reactor

**Figure 7:**
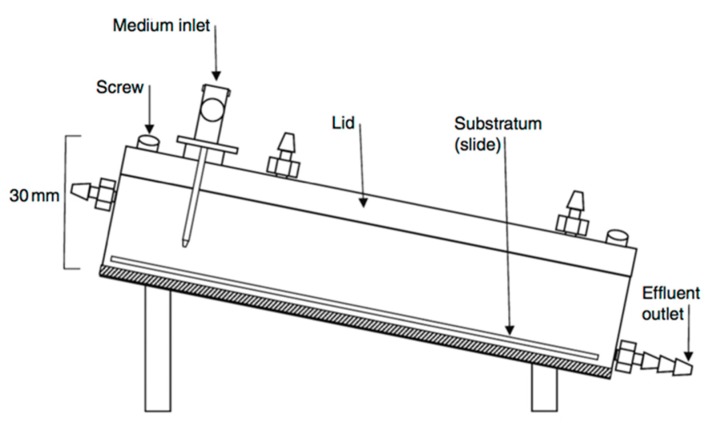
Schematic diagram of a drip flow biofilm reactor (adapted from McBain et al., 2009 [15], with permission from © 2009 Elsevier. License number: 4130791265178).

The drip flow biofilm model is often used to grow and establish solid-liquid or solid-air interface biofilms. The model usually contains four chambers in an adjustable inclined fermenter. The schematic diagram of drip-flow biofilm model is shown in Figure 7.

The biofilms grow on angled tooth surfaces, which are continuously irrigated with small volumes of fresh medium from the inlet. The incline of the fermenter enables the medium to flow over the tooth surface with biofilm, providing a low-shear environment for the biofilm.

The model allows plaque to grow on the tooth surface and to stabilize for longer periods, which enables relatively stable development of microbial communities [36]. However, as the medium flow on the surface of the substrata might not be always consistent, aerial heterogeneity over the surface of substratum may exist [15]. This model is commercially available (Biosurfaces Technologies Corporation, Bozeman, MT, USA), and thus is commonly used by researchers. This model was used to test disinfection efficacy [37], to investigate the effect of powered tooth brushing on removal of biofilm [38], and to compare the antibacterial effects of anti-caries agents [36].

#### 3.2.5. The Multiple Sorbarod Model

**Figure 8:**
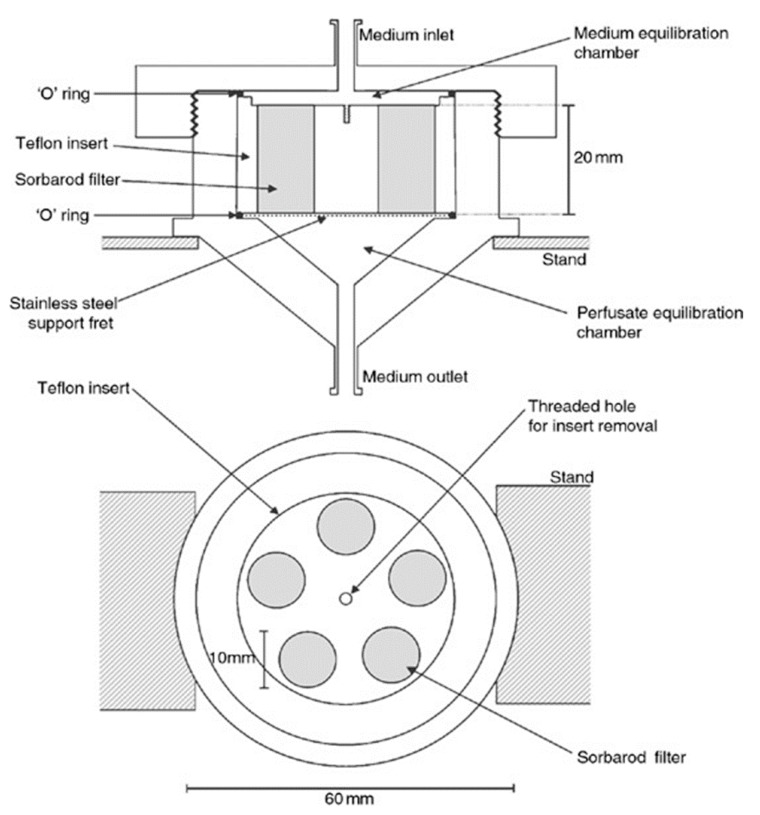
A schematic diagram of a multiple Sorbarod device (adapted from McBain et al., 2005 [41], with permission from © 2005 Wiley Online Library. License number: 4130790067572).

The multiple Sorbarod model uses a permeable Sorbarod membrane as the substratum. The fresh medium is supplied by continuous perfusion through the membrane. The exfoliated bacterial cells and metabolic wastes will be removed with spent culture medium. The schematic diagram of the multiple Sorbarod model is shown in Figure 8.

In this model, the flow rate of the medium can be controlled. Therefore, the growth rate of the biofilm is controllable [15]. The multiple Sorbarod model was used to investigate the effect of oral hygiene activities on anaerobic oral biofilms [39] and to assess the plaque-control effects of some specific enzymes [40]. An advantage of this model is that the growth rate of the biofilm can be controlled. Another advantage is that the detached bacterial cells in the spent culture medium can be studied to evaluate the biological effect of experimental treatment [36]. Since the model develops heterogeneous biofilm, it cannot be used in study design where homogeneity of the biofilm is important [15].

#### 3.2.6. The Multiple Artificial Mouth

The multiple artificial mouth (MAM) is a computer-controlled, multiple-station model. It has a more complicated construction than the models discussed above.

**Figure 9:**
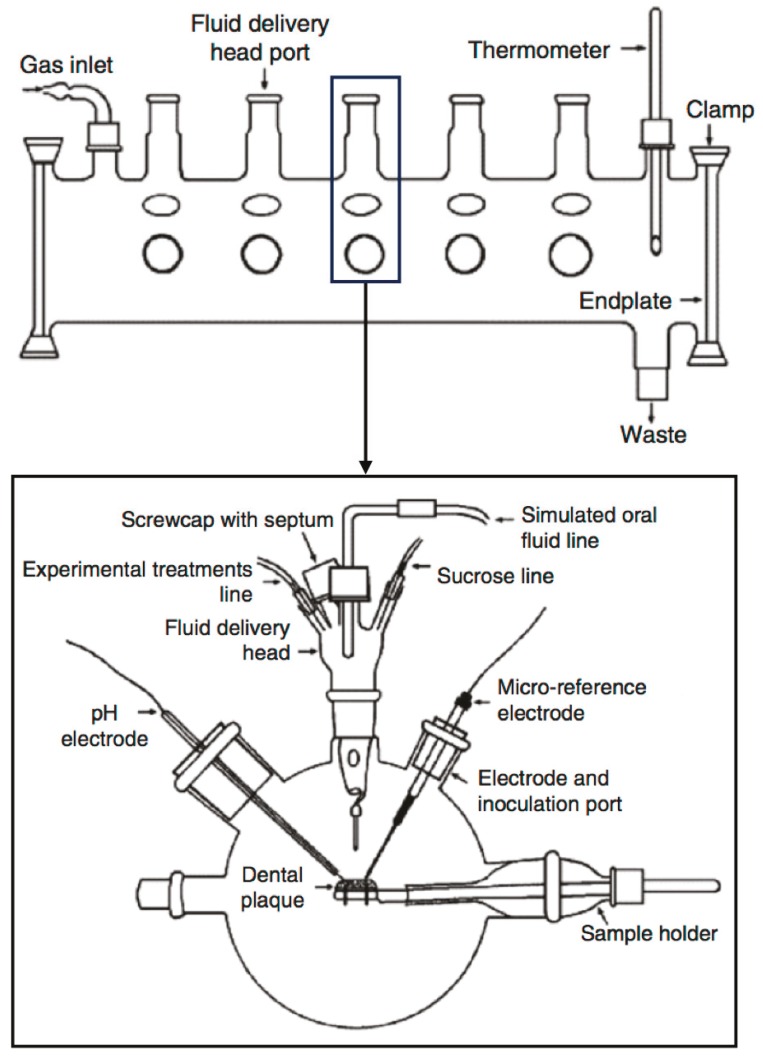
Schematic diagram of a multiple artificial mouth (adapted from Sissons et al., 2000 [47], with permission from © 2000 Springer. License number: 4130800878870).

A MAM can accurately simulate an in vivo environment using computer-controlled facilities [42]. It has several microstations, which are relatively independent to one other (Figure 9). Different experimental conditions can be applied simultaneously in different microstations.

Environmental variables can be easily controlled in the MAM. This allows analysis of the biofilm during its development, without contaminating other samples. Acidity can be monitored using a pH electrode and a micro-reference electrode [12]. These well-controlled conditions improve the standardization and flexibility of the MAM, and therefore enhance its ability to culture biofilms similar to natural oral flora. Sissons et al. found that biofilms developed in this system exhibited metabolic and pH behavior that resembled typical natural plaques [42]. The MAM has been adopted in different studies, such as biodiversity of plaques [43], fluoride and phosphate assay [44], plaque calcium level measurement [45], and the generation of consortia using major plaque species [46]. The biofilm samples in this model were exposed to the same temperature and gas-phase fluctuation. The MAM aims to mimic the oral environment. Therefore, saliva substitutes play an important role in the model. Approximate laminar flows are applied to simulate the situations in the oral cavity, instead of turbulent flow in chemostat.

## 4. Summary

Dental biofilm is an essential factor in the etiology of dental caries. Cariogenic bacteria *streptococci*, *actinomycetes,* and *lactobacilli* are found to be more closely associated with dental caries. Laboratory microbial culture models can provide a steady and controllable environment for cariology research. The models play an important role in cariology research in investigating caries pathogenicity, testing effects of new caries prevention methods, and developing new caries-preventing products. Each model has its advantages and disadvantages from both experimental design and experiment cost. Table 1 shows a comparison of the discussed in vitro biofilm systems.

The designs of the biofilm models that are included vary from simple to sophisticated according to the purposes of investigation. Agar plate and microtiter are microbial culture models in the closed system that are low-cost and simple to manage. Microbial culture models in the open system are more complex and the biofilms generated are closer to natural dental plaque. Selection of the type of model used for a biofilm study depends on the growth conditions, requirements for the specific biofilm, and purposes of the study.

## Figures and Tables

**Table 1:** Characteristics of common microbial culture models for cariology research.

Parameter	Agar Plate	Microtiter	Chemostat	Flow Cell	CDFF	Drip Flow	MSD	MAM
Duration	Hours to days	Hours to days	Hours to days	Hours to days	Days to weeks	Days to weeks	Days to weeks	Days to weeks
Planktonic phase	Controlled	Controlled	Controlled	Controlled	None	None	None	None
Growth control by media	None	Via plank-tonic phase	Yes	Yes	Yes	Yes	Yes	Yes
Fluid flow	No	No	Turbulent	Laminar	Laminar	Drop	Laminar	Drop
Shear force	No	No	Yes	Yes	Yes	No	Yes	No
Defined thickness	No	No	Achievable	Achievable	Yes	No	No	No
Timed reagents	No	Manually	Yes	Pulse	Yes	Yes	Yes	Computer control
Alternative substrate	No	Yes	Yes	Yes	Yes	Yes	No	Yes
Different conditions	No	No	No	No	No	Yes	No	Yes
Subsampling during growth	Yes	Yes	Yes	Yes	Yes	Yes	Yes	Yes

CDFF = Constant depth film fermenter; MSD = Multiple Sorbarod device; MAM = Multiple artificial mouth.
